# Activation of the Wnt/β-catenin signalling pathway enhances exosome production by hucMSCs and improves their capability to promote diabetic wound healing

**DOI:** 10.1186/s12951-024-02650-x

**Published:** 2024-06-26

**Authors:** Liming Wang, Jun Chen, Jia Song, Yingyue Xiang, Mengmeng Yang, Longqing Xia, Jingwen Yang, Xinguo Hou, Li Chen, Lingshu Wang

**Affiliations:** 1 Department of Endocrinology and Metabolism, Qilu Hospital of Shandong University, Jinan, Shandong 250012 China; 2 Institute of Endocrine and Metabolic Diseases of Shandong University, Jinan, Shandong 250012 China; 3 Key Laboratory of Endocrine and Metabolic Diseases, Shandong Province Medicine & Health, Jinan, Shandong 250012 China; 4 Jinan Clinical Research Center for Endocrine and Metabolic Diseases, Jinan, Shandong 250012 China

**Keywords:** Exosomes, Wnt/β-catenin signalling, HucMSCs, Wound healing, Exocytosis

## Abstract

**Background:**

The use of stem cell-derived exosomes (Exos) as therapeutic vehicles is receiving increasing attention. Exosome administration has several advantages over cell transplantation, thus making exosomes promising candidates for large-scale clinical implementation and commercialization. However, exosome extraction and purification efficiencies are relatively low, and therapeutic heterogeneity is high due to differences in culture conditions and cell viability. Therefore, in this study, we investigated a priming procedure to enhance the production and therapeutic effects of exosomes from human umbilical cord mesenchymal stem cells (hucMSCs). After preconditioning hucMSCs with agonists/inhibitors that target the Wnt/β-catenin pathway, we assessed both the production of exosomes and the therapeutic efficacy of the optimized exosomes in the context of diabetic wound healing, hoping to provide a safer, more stable and more effective option for clinical application.

**Results:**

The Wnt signalling pathway agonist CHIR99021 increased exosome production by 1.5-fold without causing obvious changes in the characteristics of the hucMSCs or the size of the exosome particles. Further studies showed that CHIR99021 promoted the production of exosomes by facilitating exocytosis. This process was partly mediated by SNAP25. To further explore whether CHIR99021 changed the cargo that was loaded into the exosomes and its therapeutic effects, we performed proteomic and transcriptomic analyses of exosomes from primed and control hucMSCs. The results showed that CHIR99021 significantly upregulated the expression of proteins that are associated with cell migration and wound healing. Animal experiments confirmed that, compared to control hucMSC-derived exosomes, CHIR99021-pretreated hucMSC-derived exosomes (CHIR-Exos) significantly accelerated wound healing in diabetic mice, enhanced local collagen deposition, promoted angiogenesis, and reduced chronic inflammation. Subsequent in vitro experiments confirmed that the CHIR-Exos promoted wound healing by facilitating cell migration, inhibiting oxidative stress-induced apoptosis, and preventing cell cycle arrest.

**Conclusions:**

The Wnt agonist CHIR99021 significantly increased exosome secretion by hucMSCs, which was partly mediated by SNAP25. Notably, CHIR99021 treatment also significantly increased the exosomal levels of proteins that are associated with wound healing and cell migration, resulting in enhanced acceleration of wound healing. All of these results suggested that pretreatment of hucMSCs with CHIR99021 not only promoted exosome production but also improved the exosome therapeutic efficacy, thus providing a promising option for large-scale clinical implementation and commercialization.

**Graphical Abstract:**

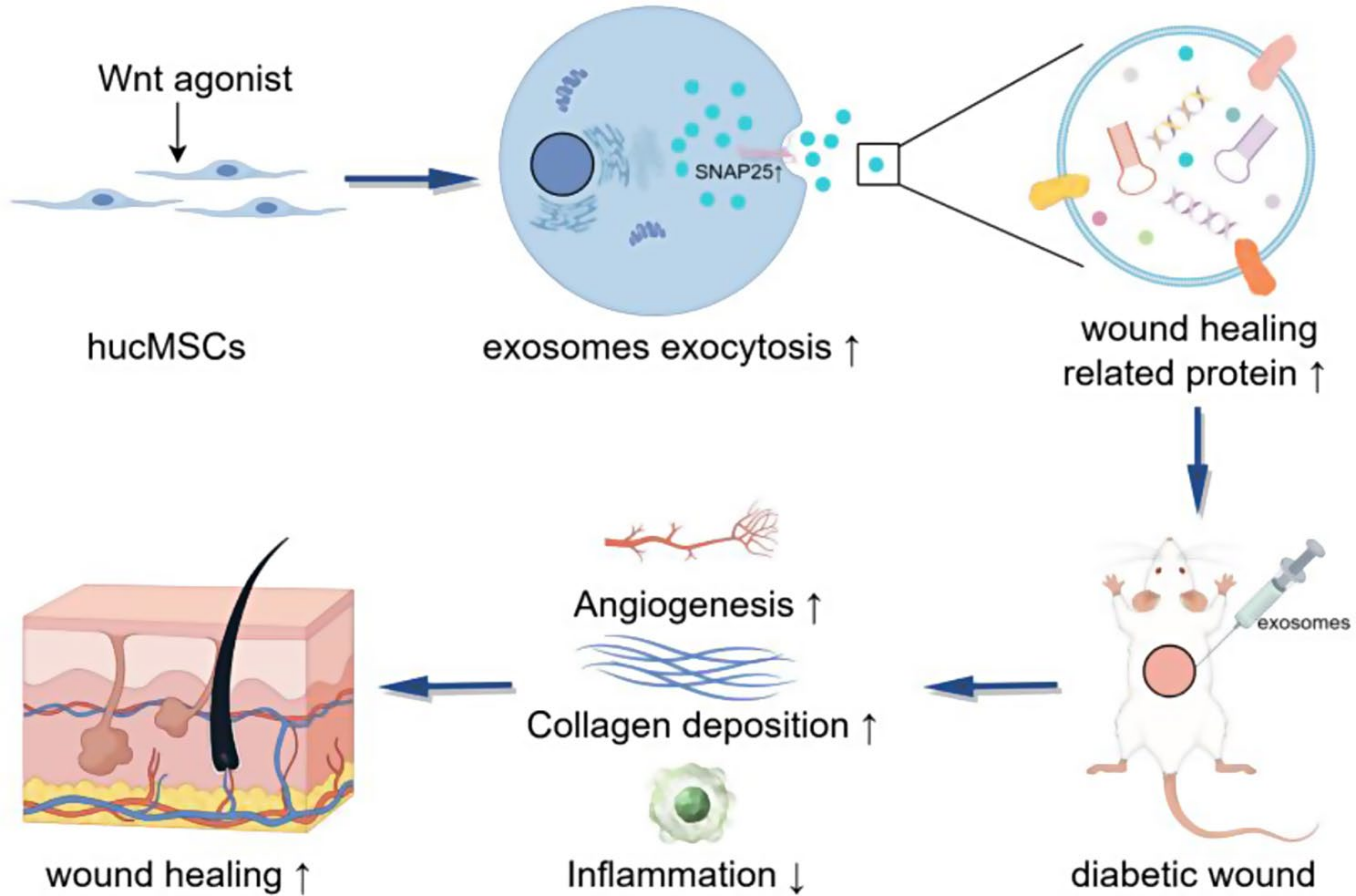

**Supplementary Information:**

The online version contains supplementary material available at 10.1186/s12951-024-02650-x.

## Background

Stem cell-related technologies have been increasingly studied in recent years [1], and human mesenchymal stem cells (MSCs) have been extensively assessed in clinical trials [2, 3]. MSCs exert their therapeutic effects through paracrine factors that are loaded into exosomes (Exos) [4]. The administration of MSC-derived exosomes (MSC-Exos) has several advantages over traditional stem cell transplantation, such as prevention of tumorigenicity, low immunogenicity, minimal contamination, and ease of long-term storage [5–7]. As a result, MSC-Exos have emerged as an attractive cell-free alternative treatment and have potential for widespread implementation as advanced therapy medicinal products (ATMPs) [8].

However, several obstacles limit the large-scale clinical application and commercialization of MSC-Exos. First, the efficiency of the currently available extraction and purification procedures is relatively low, resulting in a low input‒output ratio [9–11]. Additionally, the loading of cargo into exosomes and the secretion of exosomes are influenced by various factors, such as cell type, culture environment, cell viability and even cell passage number [12], making it challenging to ensure the consistency and efficacy of exosomes across different batches. Hence, for enhanced therapeutic efficacy and promotion of the application of MSC-Exos, measures that enhance the yield of exosomes per cell and optimize the loading of cargo within exosomes are urgently needed to guarantee improved therapeutic effects [13, 14].

Previous studies have suggested that preconditioning MSCs with various physical and chemical stimuli, such as hypoxia and lipopolysaccharide (LPS), might enhance the therapeutic effects of the corresponding MSC-Exos [15, 16]. However, these harmful stimuli may also negatively affect cell vitality and proliferation, leading to cell senescence and differentiation. The Wnt signalling pathway is a highly conserved pathway that plays crucial roles in various biological processes, including stemness maintenance, embryonic development, organogenesis, tissue regeneration, and diseases such as cancer [17]. In theory, this pathway might affect the secretome, but strong evidence of the effect of Wnt activation on cell paracrine activity is lacking. Several studies have indicated that activation of the Wnt signalling pathway can enhance the formation of multivesicular bodies (MVBs) [18] and facilitate the exocytosis of extracellular vesicles (EVs) [19–21]. Other studies have suggested that modulation of EV secretion via activation of the Wnt signalling pathway depends on the cell type [22]. The Wnt signalling pathway is known to regulate the proliferation and maintenance of stemness of MSCs; however, few, if any, studies have focused on whether this pathway can increase exosome production by MSCs or affect the loading of cargo into these vesicles.

Therefore, in this study, we aimed to explore a priming procedure to enhance exosome production by human umbilical cord MSCs (hucMSCs), which are readily available and highly viable cells that are widely used in stem cell therapy, as well as the effects of these exosomes. We used two agonists and one inhibitor to evaluate the effect of the Wnt signalling pathway on the production of exosomes by hucMSCs. Nanoparticle tracking analysis (NTA) was used to determine the size and amount of secreted exosome particles, while cell transcriptomics and exosomal proteomics were used to screen the enriched pathways and enhanced functions. Animal model and functional verification experiments were chosen and carried out according to the changes in the secretome revealed by proteomics. Through these investigations, we hoped to provide a safe, stable and effective priming strategy to enhance exosome production and optimize the therapeutic effects of exosomes, thus facilitating the clinical implementation and commercialization of MSC-Exos.

## Methods

### Cell culture

Wharton’s jelly from the umbilical cord was sectioned into 1 mm pieces, which were subsequently cultured in α-MEM (Gibco, USA) supplemented with 10% foetal bovine serum (Gibco, USA), 100 U/mL penicillin, and 100 µg/mL streptomycin (Gibco, USA). Passaging was carried out once the cells achieved 80–90% confluence. NIH/3T3 cells were cultured in DMEM (Gibco, USA) supplemented with 10% neonatal bovine serum, 100 U/mL penicillin, 100 µg/mL streptomycin, 1% sodium pyruvate, 1% nonessential amino acids, and 1% glutamine at 37 °C in 5% CO₂. The MSCs were identified as previously described [23]. Flow cytometry analysis was also conducted to characterize the hucMSCs based on their expression of CD105 (Biogems, USA) and CD73 (Biogems, USA) and the lack of expression of CD34 (Biolegend, USA) and HLA-DR (Biolegend, USA). The ability of hucMSCs to differentiate into adipocytes and osteoblasts was assessed using Oil Red O (Solarbio, China) staining for lipid droplets and Alizarin Red S staining (Solarbio, China) for calcium nodes. We used two Wnt signalling pathway agonists, namely, CHIR99021 (Selleck Chemicals, USA) and CP21R7 (Selleck Chemicals, USA), and the Wnt signalling pathway inhibitor XAV939 (Selleck Chemicals, USA). CHIR99021 inhibits GSK-3α and GSK-3β, CP21R7 inhibits GSK-3β, and XAV939 promotes the degradation of β-catenin by stabilizing the limiting concentrations of axin and other factors that are involved in its degradation. hucMSCs were stimulated with 5 µM CP21R7, 6.25 µM CHIR99021 and 10 µM XAV939. NIH/3T3 cells were stimulated with 800 µM hydrogen peroxide (Maokangbio, China).

### Exosome isolation and identification

When the hucMSCs reached 70 ~ 80% confluence, the cells were cultured in serum-free medium for 48 h, and the cell supernatants were collected. The cell supernatants were first centrifuged at 10,000×g for 1 h to remove cellular debris and large vesicles and subsequently centrifuged at 120,000×g for 70 min to obtain exosomes, which were resuspended in PBS. The sizes of the exosomes were determined via NTA (PARTICLE METRIX, Germany). Exosome morphology was determined by transmission electron microscopy (TEM; FEI, Tecnai G2 Spirit BioTwin, USA). The expression levels of CD9, CD63, TSG101 and calnexin were assessed via Western blotting.

### Western blotting

Total proteins were extracted and separated via sodium dodecyl sulphate‒polyacrylamide gel electrophoresis (SDS‒PAGE) and subsequently transferred to PVDF membranes (IPVH00010 0.45 μm, Millipore, USA). The membranes were blocked with 5% skim milk for 1 h at room temperature and incubated with specific primary antibodies at 4 °C overnight. Then, the cells were incubated with HRP-conjugated secondary antibodies for 1 h at room temperature. Primary antibodies against the following target proteins were used: β-actin (1:5000, AB0035), Rab27a (1:1000, Proteintech 17817-1-AP), Rab7 (1:1000, ABclonal A12308), Alix (1:2000, Proteintech 12422-1-AP), TSG101 (1:1000, Abcam ab125011), CD63 (1:1000, Abcam ab134045), CD9 (1:1000, Abcam ab263019), HSP70 (1:1000, Abcam a181606), VAMP2 (1:1000, ABclonal A4235), VAMP3 (1:10000, Proteintech 10702-1-AP), SNAP25 (1:5000, Proteintech 60,159-Ig), β-catenin (1:10000, Abcam ab32572), LAMP2 (1:10000, ABclonal A22482), STXBP1 (1:1000, Proteintech 11459-1-AP), AKT (1:1000, CST 9272S), p-AKT (1:1000, CST 4060S), p38 (1:1000, CST 9212S), p-p38 (1:1000, CST 9211S), caspase 3 (1:1000, CST 9662S), and cleaved caspase 3 (1:1000, CST 9661S).

### Acetylcholinesterase (AChE) enzyme-linked immunosorbent assay (ELISA) and NTA

The concentrations of AChE in the hucMSC culture supernatants were determined using an AChE ELISA kit (MLbio, China) following the manufacturer’s instructions, and the results were normalized according to the total hucMSC protein concentrations. The total protein concentrations were determined by a BCA kit (Epizyme, China). NTA was performed using ZetaView NTA and its corresponding software (ZetaView 8.05.14). Then, the hucMSCs were digested with trypsin, the number of cells was counted with a cell counter, and the concentration of exosomes was normalized to the number of hucMSCs.

### RNA-seq

Total RNA was isolated using TRIzol reagent (Invitrogen, USA) following the manufacturer’s procedure. Library construction and sequencing were performed at Shenzhen BGI Genomics Co., Ltd. DESeq2 (v1.28.1) was applied to perform the differential expression analysis. Kyoto Encyclopedia of Genes and Genomes (KEGG) pathway enrichment was performed with ClusterProfiler (v3.10.1).

### Proteomics

Proteomics was performed at Shanghai Jingtian Biotechnology Co., Ltd. Raw DIA data were processed and analysed by Spectronaut 17 (Biognosys AG, Switzerland) with default settings. Decoy generation was set to mutated, which is similar to scrambled but applies only a random number of AA position swamps (min = 2, max = length/2). The normalization strategy was set to local normalization. Peptides that passed the 1% Q value cut-off were used to calculate the major group quantities via the MaxLFQ method.

### RNAi

RNAi was performed via the transfection of siRNA oligos using Lipofectamine 2000 reagent (Invitrogen, USA) according to the manufacturer’s instructions. The three pairs of SNAP25 siRNA sequences used were as follows: sense1, 5’-GUCCUUGUAACAAGCUUAATT-3’; antisense1, 5’-UUAAGCUUGUUUACAAGGACTT-3’; sense2, 5’-CCGCAGGGUAACAAAUGAUTT-3’; antisense2, 5’-AUCAUUUGUUUACCCUGCGGGTT-3’; sense3, 5’-GUGUCGAAGAAGGCAUGAATT-3’; and antisense3 5’-UUCAUGCCUUCUUCUUCGACACTT-3’. The sequences of the negative control (NC) siRNAs used were as follows: sense 5’- UUCUCCGAACGUGUCACGUTT-3’ and antisense 5’-ACGUGACACGUUCGGGAGAATT-3’.

### Exosome tracing

NIH/3T3 cells were seeded in 24-well plates, and when the cell confluence reached approximately 80%, four groups of exosomes labelled with DIO (Invitrogen, USA) were separately added and incubated for 24 h. The cytoskeleton was stained with rhodamine-labelled phalloidin (ABclonal, China). After 4′,6-diamidino-2-phenylindole (DAPI) staining, images were captured via fluorescence microscopy.

### Cell migration

NIH/3T3 cells (2 × 10⁵) were plated in 6-well plates, and when the cell confluence reached 100%, wounds with smooth edges were generated by scratching with 200 µL pipette tips. Serum-free DMEM supplemented with exosomes (50 µg/mL) or PBS was added to the wells. Wound healing was observed at 0, 6, 12, and 24 h. The degree of healing was calculated with the following formula: Healing area = (${A}_{0}$-${A}_{n}$)/${A}_{0}$ *100, where ${A}_{0}$ is the initial scratch area and ${A}_{n}$ is the scratch area at the measurement time point.

### Animals

The animal experiments were approved by the Animal Ethics Committee of Qilu Hospital, Shandong University (IACUC Issue No. DWLL-2023-092). BALB/c mice were purchased from SPF Biotechnology Co., Ltd. (Beijing). The mice were housed in a controlled environment with a 12 h light/dark cycle at a temperature of 22–25 °C and 55% ± 5% humidity. Eight-week-old BALB/c mice were fasted for 12 h and then injected with streptozotocin (STZ, 150 mg/kg, Aladdin, China) to establish a type 1 diabetes mellitus (T1DM) model. Diabetes was defined as a fasting glucose level ≥ 16.7 mmol/L in two consecutive measurements. For generation of stable diabetic mice, the mice were reared for 4 weeks, and blood glucose was measured again before wound formation. The mice were anaesthetised with isoflurane, and a circular wound with a diameter of 8 mm was created on their backs. Twenty mice were randomly divided into four groups: the CTRL group, DM group, DM + CTRL-Exo group and DM + CHIR-Exo group. The mice were injected with exosomes (200 µg of exosomes in 200 µL of PBS) or 200 µL of PBS at 4 points around the wound (injections once every 7 days). Wound healing was recorded on days 0, 3, 7, 10, and 14 by capturing images with the same camera, and a metal ruler was placed next to the wound to indicate the wound size. The wound healing area in the photographs was quantified using ImageJ.

### Histology

Skin tissues were fixed with 4% paraformaldehyde for 72 h, embedded in paraffin, and sectioned at a thickness of 5 μm. Sections were deparaffinized, hydrated, and stained with haematoxylin-eosin (H&E) and Masson’s trichrome staining kits (Servicebio, China).

### Immunohistochemistry

Sections were deparaffinized, hydrated and subjected to antigen retrieval using citrate buffer (pH = 6; Servicebio, China). Then, the cells were treated with 3% hydrogen peroxide to inactivate endogenous peroxidases for 15 min and blocked at room temperature for 30 min in a protein-blocking solution (10% normal goat serum). Primary antibodies were added, and the samples were incubated at 4 °C overnight. The sections were then incubated with secondary antibodies at room temperature for 60 min and stained with 3,3′‐diaminobenzidine (DAB) solution (Genetech, GK600505, China). Images were captured using a microscope (Olympus BX53, Japan). The positively stained area was analysed using Image-Pro Plus software. Primary antibodies against the following target proteins were used: COL1 (1:100, ABclonal A22090), COL3 (1:500, Proteintech 22734-1-AP), CD31 (1:2000, Abcam ab182981), and IL-1β (1:100, Abcam ab283818).

### Immunofluorescence

The cells were cultured on a microscope cover glass. After fixation in 4% paraformaldehyde and permeabilization in 0.1% Triton X-100, nonspecific binding sites were blocked. Primary antibodies were added, and the samples were incubated overnight at 4 °C. Then, secondary antibodies, including DyLight 594-conjugated goat anti-mouse IgG (1:400; Abbkine, China) or DyLight 488-conjugated goat anti-rabbit IgG (1:400; Abbkine, China), were added and incubated at 37 °C for 1 h. Then, DAPI staining was performed. Images were captured using an LSM980 confocal scanning microscope (Zeiss, Jena, Germany). Primary antibodies against the following target proteins were used: SNAP25 (1:100; Proteintech 60159-1-Ig), VAMP3 (1:100; Proteintech 10702-1-AP), LAMP2 (1:200; ABclonal a22482), CD63 (1:200; Proteintech 25682-1-AP), and Rab7a (1:200; ABclonal a12308).

### Statistical analysis

All the data are presented as the mean ± SEM. Differences between the groups were evaluated using an unpaired Student’s t test or one-way ANOVA followed by Tukey’s test via GraphPad Prism 8 software (8.4.2). *p* < 0.05 was considered to indicate a statistically significant difference.

## Results

### The characteristics of hucMSCs and Exosomes remained unchanged after Wnt signalling priming

**Fig. 1:**
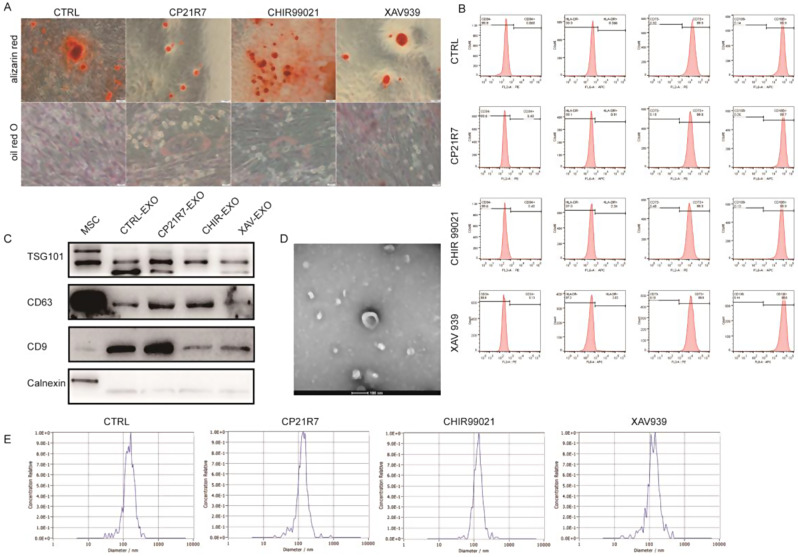
The characteristics of hucMSCs and exosomes remained unchanged after Wnt signalling priming. (**A**) Alizarin red and oil red O staining after treatment of hucMSCs with Wnt agonists or inhibitors (**B**) Flow cytometry identification of hucMSCs maker after treatment of hucMSCs with Wnt agonists or inhibitors. (**C**) Western blot analysis of exosome marker from different pretreated hucMSCs. (**D**) Exosome transmission electron microscopy. (**E**) Exosome NTA assay

Before examining the impact of the Wnt signalling pathway on the production and cargo loading of exosomes, we had to verify that the properties of hucMSCs and exosomes after Wnt signalling priming were unaltered. In this study, 2 Wnt agonists (CHIR99021 and CP21R7) and 1 inhibitor (XAV939) were tested. After treatment, the ability of the pretreated hucMSCs to differentiate into adipocytes or osteocytes did not significantly change compared to that of the untreated hucMSCs, suggesting that altered Wnt signalling had no obvious effect on the differentiation potential of MSCs (Fig. 1A). In addition, flow cytometry analysis revealed that the hucMSCs subjected to different treatments exhibited comparable negative CD34 and HLA-DR expression and positive CD105 and CD73 expression, suggesting that these procedures did not significantly change the surface marker profile of the MSCs (Fig. 1B). Furthermore, to evaluate cell senescence, we cultured hucMSCs with or without CHIR99021 pretreatment for 7 continuous passages. By the 7th passage, the β-galactosidase-positive area was significantly reduced by priming with CHIR99021 compared to that in the untreated hucMSCs, suggesting that CHIR99021 delayed the senescence of hucMSCs (Figure S1A).

Exosomes were then isolated from hucMSCs that were subjected to different pretreatment procedures and characterized via Western blotting analysis. The results revealed the presence of TSG101, CD63, and CD9 in the exosomes from all four groups, while calnexin, which is a negative marker of exosomes, was absent (Fig. 1C). TEM analysis confirmed the characteristic double-membrane structure that is commonly observed in exosomes (Fig. 1D). Finally, the size distribution of the exosome particles was determined using NTA analysis, and the results indicated an average particle size of approximately 130 nm in the exosomes of all four groups (Fig. 1E). These findings collectively indicated that the administration of Wnt signalling pathway agonists or inhibitors does not exert any discernible effect on the characteristics of MSCs or the inherent properties of the secreted exosomes.

### Wnt agonist treatment increased hucMSC exosome production

**Fig. 2:**
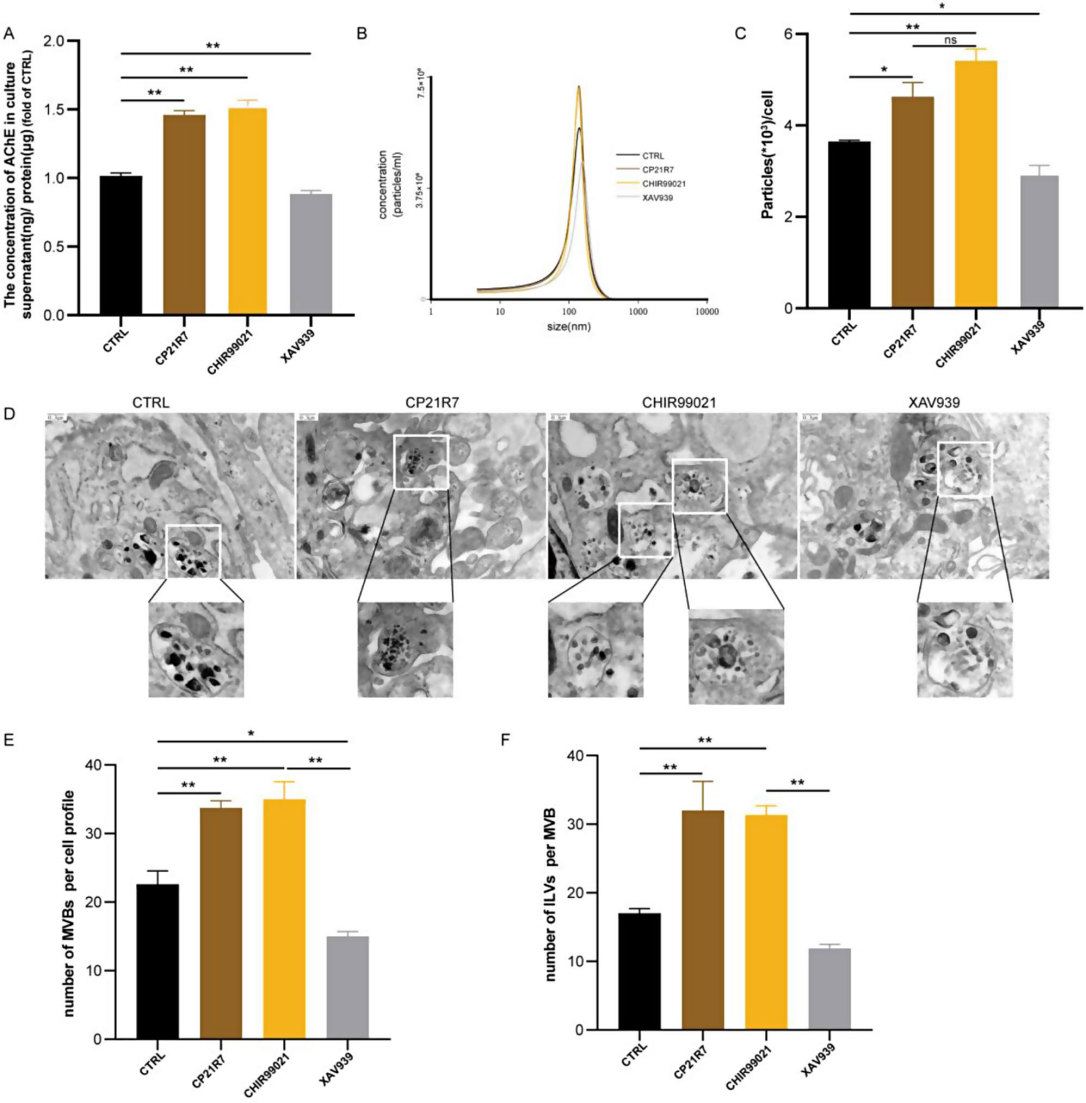
Wnt agonist treatment increased hucMSC exosome production. (**A**) ELISA detection of AChE in hucMSC supernatants from different treatment groups (**B**) Exosome particle size distribution. (**C**) Quantification of different treatment groups of exosomes by NTA. (**D**-**F**) Representative TEM images of hucMSCs and the quantitation analysis on the MVBs and ILVs after hucMSCs were treated with Wnt agonists or inhibitors. Data are represented as mean ± SEM. *P < 0.05, **P < 0.01

Subsequently, we investigated the potential impact of the Wnt signalling pathway on exosome production in hucMSCs. Previous studies demonstrated a positive correlation between exosome content and AChE levels in culture supernatants [24]. Therefore, we initially quantified the AChE concentration in the MSC supernatant as an indicator of exosome content. To ensure accurate comparisons, we normalized the results according to the total protein content. A 1.5-fold increase in AChE levels was observed in the groups treated with Wnt signalling pathway agonists, whereas inhibition of this pathway led to a decrease in exosome secretion (Fig. 2A). To further verify this trend, we employed NTA to quantify the exosome size and number. The exosome number was normalized according to the number of MSCs. The NTA results proved that treatment with Wnt agonists resulted in a similar 1.5-fold increase in exosome secretion (Fig. 2C). Notably, this increase in secretion did not significantly influence the particle size of the exosomes (Fig. 2B). We also found that the effect of the agonist CHIR99021 was slightly more pronounced than that of CP21R7 after the results were normalized to the cell number, but the difference was not statistically significant (Fig. 2B-C). Additionally, we utilized TEM to visualize the intracellular content of the MVBs and intraluminal vesicles (ILVs). Our observations revealed an increase in the intracellular MVBs and ILVs following treatment with a Wnt signalling pathway agonist (Fig. 2D-F). Collectively, the above results suggested that Wnt signalling pathway agonists can increase the content of MVBs and ILVs within hucMSCs and increase exosome secretion by approximately 1.5-fold. Nevertheless, considering the consistency of the results, we chose CHIR99021 as the Wnt agonist for use in the subsequent proteomic, transcriptomic and functional validation experiments.

### CHIR99021 increased exosome secretion by promoting SNAP25-dependent exocytosis

Our aforementioned results suggested that Wnt signalling pathway agonists can increase exosome production by 1.5-fold. Previous studies revealed that exosome formation is affected mainly through the following three key processes: MVB and ILV production, lysosome-mediated degradation, and exocytosis.

**Fig. 3:**
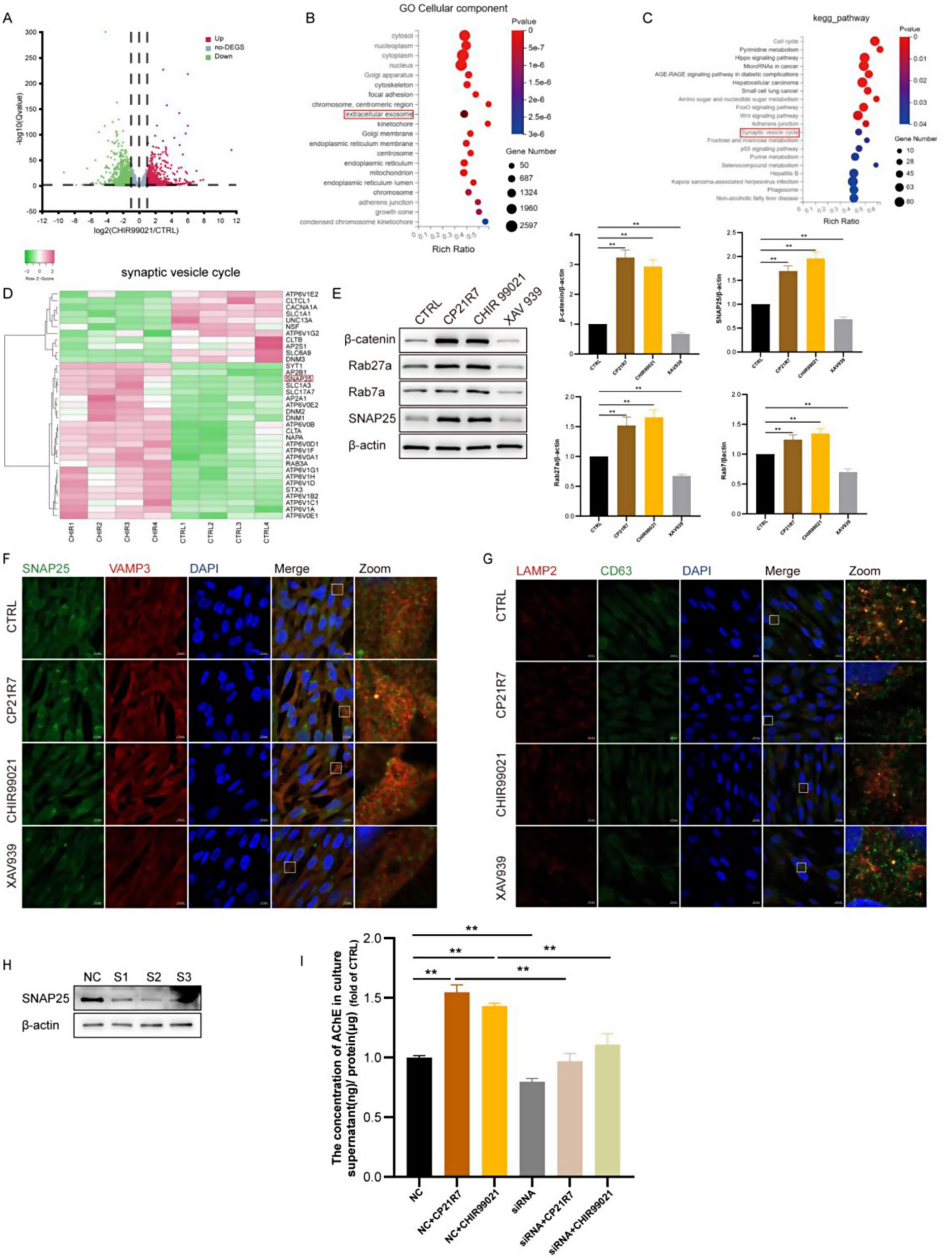
CHIR99021 increased exosome secretion by promoting SNAP25-dependent exocytosis. (**A**) Volcano plot showing differential gene expression of hucMSCs in the CHIR99021-treated group versus the control group. (**B**) GO enrichment analysis of hucMSCs in CHIR99021-treated group versus control group. (**C**) KEGG enrichment analysis of hucMSCs in CHIR99021-treated group versus control group. (**D**) Heatmap of gene clustering of hucMSCs transcriptomics in synaptic vesicle cycle between CHIR99021-treated and control groups. (**E**) Western blot analysis of β-catenin, Rab27a, Rab7, SNAP25 in hucMSCs treated with Wnt signaling pathway agonists and inhibitors. (**F**) Co-location of SNAP25 with VAMP3 was observed using immunofluorescence after treatment of hucMSCs with Wnt signaling pathway agonists and inhibitors. (**G**) Co-location of LAMP2 with CD63 was observed using immunofluorescence after treatment of hucMSCs with Wnt signaling pathway agonists and inhibitors. (**H**) Western blot analysis of SNAP25 knockdown efficiency. (**I**) After knocking down of SNAP25, hucMSCs were treated with Wnt signaling pathway agonists for 48 h, supernatants were collected, and AChE concentration was analysis by ELISA. Data are represented as mean ± SEM. *P < 0.05, **P < 0.01

To investigate the underlying mechanisms driving this enhanced secretion, we conducted transcriptomic analysis of hucMSCs treated with or without CHIR99021. Differentially up- and downregulated genes were revealed by volcano plot analysis (Fig. 3A). Subsequent Gene Ontology (GO) analysis indicated that the differentially expressed genes were enriched in the term “extracellular exosomes” (Fig. 3B). Furthermore, KEGG analysis revealed potential enrichment in the “synaptic vesicle cycle” pathway, which is a crucial pathway involved in cytosolic exocytosis (Fig. 3C). By conducting a clustering heatmap analysis of the 35 genes associated with the synaptic vesicle cycle, we found that 24 genes were upregulated in the CHIR99021 group. Notably, one of the upregulated genes was SNAP25, an essential constituent of the soluble N-ethylmaleimide-sensitive factor attachment protein receptor (SNARE) complex that plays a critical role in exocytosis processes (Fig. 3D). To validate this result, we conducted Western blotting analysis and observed a similar upregulation of SNAP25 protein expression induced by CHIR99021. Additionally, CHIR99021 upregulated the expression of exocytosis regulatory proteins, namely, Rab27a and Rab7a. Rab27a promotes the accumulation and transport of MVBs to the plasma membrane, while Rab7a mediates MBV maturation and transport and endosome-lysosome fusion. Conversely, treatment with the Wnt signalling inhibitor XAV939 had the opposite effect (Fig. 3E). Notably, no changes were observed in the protein levels of other SNARE complex components, including STXBP1 VAMP2 and VAMP3, in the expression of the ESCRT components ALIX and TSG101 or in the lysosome marker LAMP2 (Figure S1B). Taken together, these results demonstrated that Wnt signalling regulates the exocytosis of hucMSCs.

Next, we utilized TEM and confocal microscopy to visualize the intracellular localization and content of MVBs and ILVs. Considering that SNAP25 interacts with its ligand VAMP3 to trigger membrane fusion and exosome release, we first examined the colocalization of SNAP25 and VAMP3. SNAP25 and VAMP3 were significantly colocalized near the plasma membrane in the Wnt agonist-treated group (Fig. 3F), suggesting that the Wnt agonist increased SNAP25-mediated membrane fusion. Additionally, RAB7a, which mediates MBV maturation and transportation, exhibited increased fluorescence enrichment at the cytosolic membrane after agonist treatment (Figure S1C). Regarding lysosome-mediated degradation, the colocalization of the lysosome marker LAMP2 with the MVB marker CD63 was reduced after Wnt agonist treatment, suggesting that the lysosome-mediated degradation of MVBs might be suppressed by the Wnt agonist (Fig. 3G). These findings were consistent with our TEM results, which showed an increase in the number of intracellular MVBs and ILVs after agonist priming (Fig. 2D-F). No significant changes were observed in the levels of exosome synthesis-related proteins, such as ALIX and TSG101 (Figure S1B). The Wnt agonists may have increased the MVB and ILV contents by suppressing lysosome-mediated degradation. However, we cannot exclude the possibility that Wnt agonists might regulate the generation of MVBs and ILVs through mechanisms that are independent of ALIX and TSG101, which needs further verification. We will only focus on the mechanism validated by transcriptomics, namely, the SNAP25-mediated exocytosis pathway.

To demonstrate whether the regulatory effect of Wnt agonists on exocytosis is SNAP dependent, we used siRNA to knock down SNAP25 (Fig. 3H). Given the consistent results observed in Fig. 2 between the AChE level analysis and NTA, we measured AChE levels as an indicator of exosome content. Notably, suppression of SNAP25 expression resulted in a significant reduction in AChE levels, and the pro-exocytosis effects of the Wnt agonists were nearly abolished (Fig. 3I). These findings suggested that Wnt signalling pathway agonists can potentially facilitate exosome release, and the underlying mechanism is partially dependent on SNAP25.

### CHIR99021 enhanced the loading of wound healing-related proteins into exosomes

**Fig. 4:**
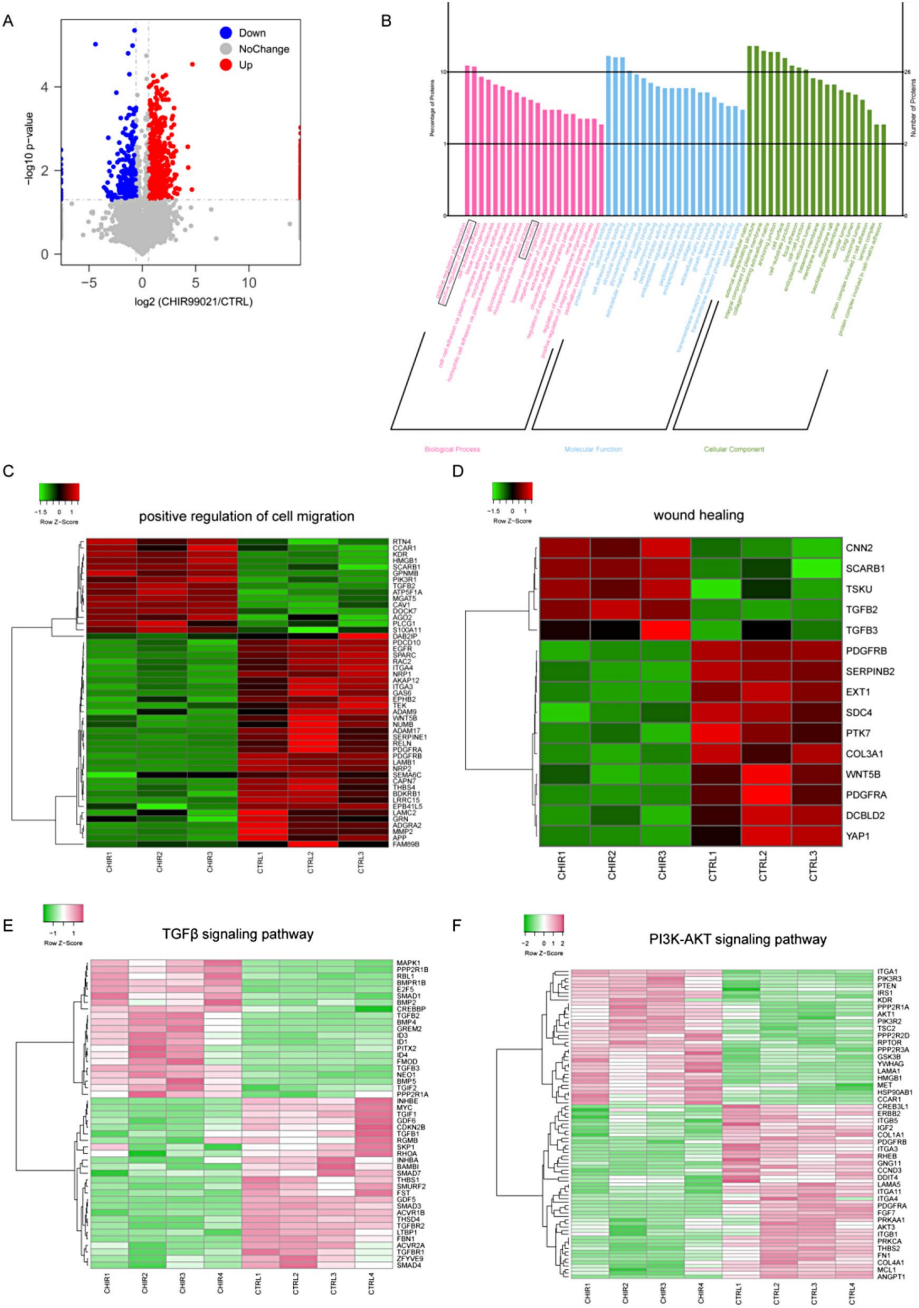
CHIR99021 enhanced the loading of wound healing-related proteins into exosomes. (**A**) Volcano plot showing differential protein of exosome between CHIR99021-treated and control groups. (**B**) GO enrichment analysis between CHIR99021 treatment group and control group. (**C**-**D**) Heatmap showing differential protein between CHIR99021 treatment group and control group. (**E**-**F**) Heatmap of hucMSCs transcriptomics clustering in CHIR99021-treated group vs. control group

Based on the above results, Wnt agonists, particularly CHIR99021, significantly increased SNAP25-mediated exosome secretion. However, importantly, an increase in exosome production alone cannot guarantee therapeutic efficacy. Therefore, we aimed to explore the impact of Wnt agonists on the cargo content of exosomes by conducting proteomics analysis of exosomes that were released by hucMSCs with or without CHIR99021 treatment (referred to as CHIR-Exos and CTRL-Exos, respectively). The volcano plot shows the differentially upregulated and downregulated proteins in response to CHIR99021 treatment (Fig. 4A). GO analysis revealed that the proteins exhibiting altered expression were enriched in two biological processes, namely, “positive regulation of cell migration” and “wound healing” (Fig. 4B). A clustered heatmap of the proteins enriched in these processes highlighted the upregulation of TGFβ2, TGFβ3, AGO2, CCAR1 and HMGB1, which are known to promote wound healing and cell migration (Fig. 4C-D). Transcriptomic analysis of hucMSCs revealed similar results, with differentially expressed mRNAs clustering in the TGFβ pathway and PI3K-Akt pathway, both of which play important roles in regulating wound healing (Fig. 4E-F). These results indicated that CHIR99021 treatment could alter the contents of exosomes and promote the enrichment of proteins that are associated with wound healing and cell migration.

### CHIR-Exos promoted diabetic cutaneous wound healing more strongly than untreated hucMSC exosomes

**Fig. 5:**
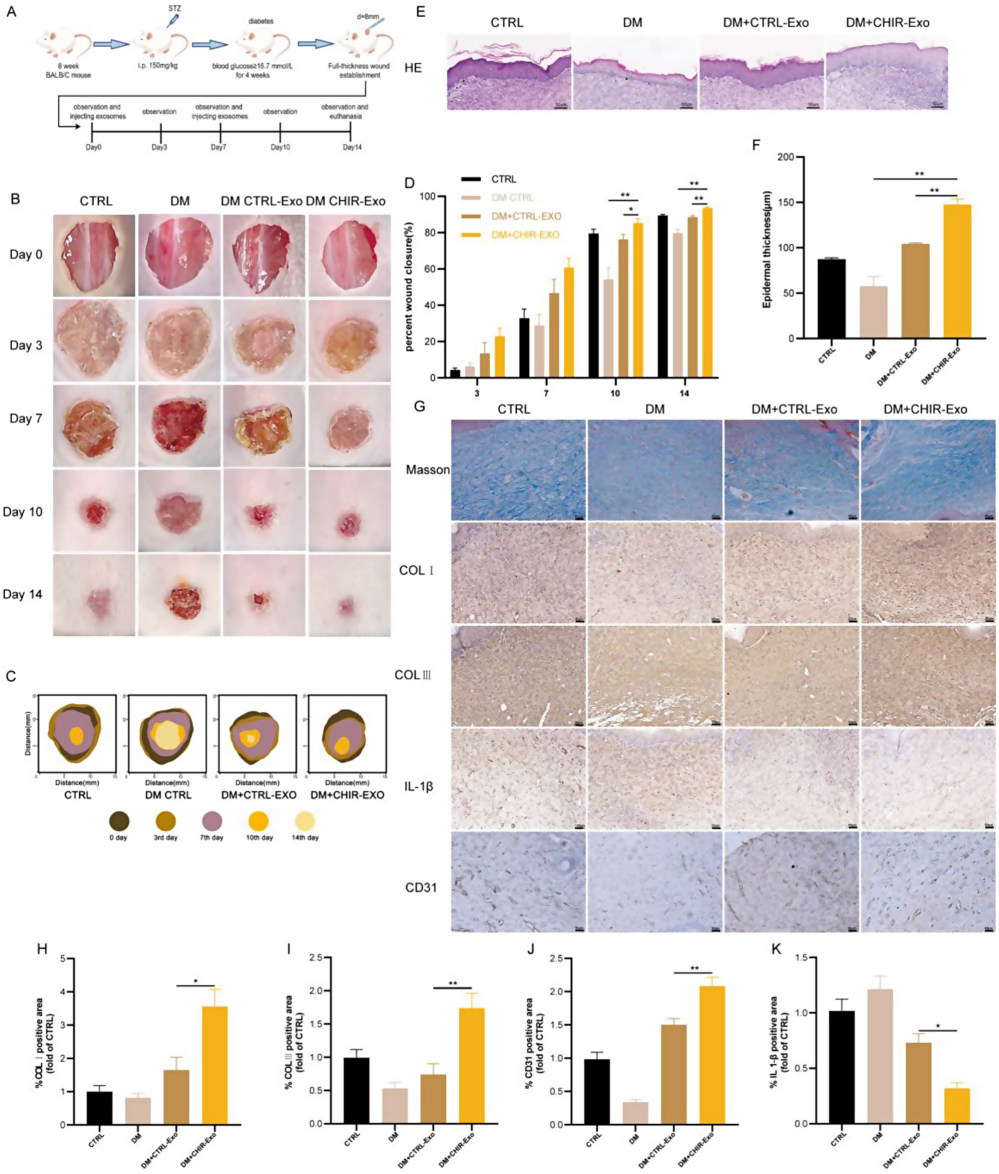
CHIR-Exos promoted diabetic cutaneous wound healing more strongly than untreated hucMSC exosomes. (**A**) Flow chart of the animal experiment. (n = 5 in each group) (**B**) Skin wound healing in 4 groups of mice on days 0, 3, 7, 10 and 14. (**C**) Pattern diagrams of skin healing in the 4 groups of mice, with coloured areas showing the area of unhealed wounds at different observation times. (**D**) Statistics on the percentage of wound healing area of mice. (**E**-**F**) H&E staining to observe the epidermal thickness of the skin at the wound in each group of mice. (**G**-**K**) Masson staining and immunohistochemical analysis of collagen deposition, IL-1β, CD31 expression in the skin of mice in each group. Data are represented as mean ± SEM. *P < 0.05, **P < 0.01

To further confirm whether the CHIR-Exos could promote wound healing in vivo, we established a full-thickness cutaneous wound model in diabetic mice (Fig. 5A). Subsequent imaging on days 0, 3, 7, 10, and 14 revealed that CHIR-Exos significantly accelerated the healing of diabetic wounds, surpassing the healing rate induced by control exosomes (referred to as CTRL-Exos). Notably, the CHIR-Exo group achieved near-complete wound closure by the 14th day of observation (Fig. 5B-D).

Histological analysis of skin sections from wounds on day 14 revealed a notable increase in the epidermal layer in the CTRL-Exo treatment groups. Remarkably, this increase was even more pronounced in the CHIR-Exo treatment group, in which the cells were arranged in a more normal pattern (Fig. 5E-F). Since Exos have been reported to engage in and enhance nearly all stages of wound healing [25], immunohistochemical staining and Masson staining were performed to assess collagen deposition, the inflammatory response, and angiogenesis in wounds. The results indicated that the deposition of both collagen I and collagen III was enhanced in the wounds of the CHIR-Exo group, and this change was accompanied by significantly reduced IL1β expression and enhanced angiogenesis at the wound site (Fig. 5G-K). These findings indicated that the administration of CHIR-Exos could enhance collagen deposition, suppress the inflammatory response and facilitate angiogenesis in diabetic wounds, thereby promoting the process of wound healing.

### CHIR-Exos promoted fibroblast migration and proliferation and suppressed apoptosis in vitro

**Fig. 6:**
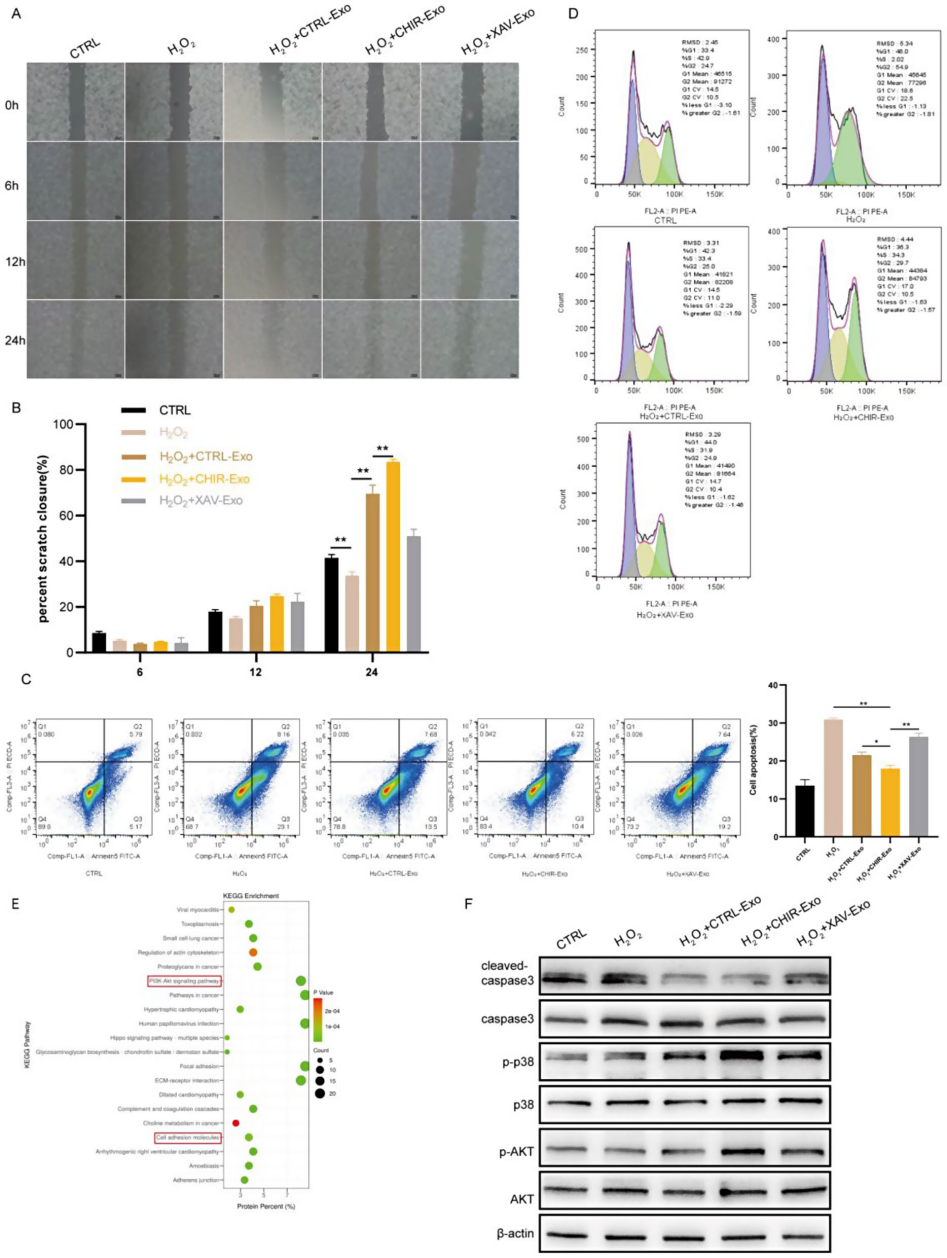
CHIR-Exos promoted fibroblast migration and proliferation and suppressed apoptosis in vitro. (**A**-**B**). Migration of NIH/3T3 cells at 0, 6, 12, and 24 h in scratch assay. (**C**) Apoptosis was detected in each group using Annexin5/PI staining, and the histogram showed the percentage of both Annexin5 (+) PI (−) and Annexin5 (+) PI (+) cells. (**D**) Cell cycle was detected using PI staining. (**E**) KEGG enrichment analysis of exosome between CHIR99021-treated and control groups. (**F**) Western blot analysis of AKT, p-AKT, p38, p-p38, caspase3 and cleaved-caspase3 in each group. Data are represented as mean ± SEM. *P < 0.05, **P < 0.01

We further verified the pro-healing effects of CHIR-Exos in the NIH/3T3 cell line. Different exosome treatments were administered to stimulate cell migration, validating the effects of exosomes from various groups on this process. The uptake of all three types of exosomes by HIN/3T3 cells was confirmed (Figure S1D). Notably, both the CTRL-Exos and CHIR-Exos significantly promoted HIN/3T3 cell migration, with the CHIR-Exos exhibiting a stronger effect than the CTRL-Exos. Conversely, XAV939-pretreated exosomes (referred to as XAV-Exos) exerted the weakest promotion of cell migration (Fig. 6A-B). Annexin V/PI staining demonstrated that the CHIR-Exos inhibited apoptosis (Fig. 6C), while the cell cycle assay revealed that these exosomes also increased the proportion of cells in the S and G2 phases, thereby promoting cell proliferation (Fig. 6D). Consistent with the Annexin V/PI staining results, CHIR-Exo treatment also decreased the protein level of cleaved caspase 3 (Fig. 6F) as well as the activation of the PI3K/AKT signalling pathway and the P38MAPK signalling pathway. KEGG analysis revealed similar results; namely, the differentially expressed proteins in the CHIR-Exos were significantly enriched in the PI3K/AKT signalling pathway and cell adhesion molecules (Fig. 6E). These findings indicated that the CHIR-Exos promoted cell migration, suppressed oxidative stress-induced apoptosis, and prevented cell cycle arrest in vitro.

**Discussion**.

MSCs are a promising therapy for various diseases ranging from diabetes to wound healing [26, 27]. Exosomes are small EVs with a size of 30–150 nm and exert most of the therapeutic effects of MSCs. Exosomes transport a diverse range of molecules, including proteins, mRNAs, miRNAs, and lipids, and thus play crucial roles in various biological processes, such as intercellular communication, immunomodulation, tumour metastasis and tissue regeneration [28–30]. However, the poor scalability of their production has limited the large-scale application of MSC-derived exosomes in the clinic. Therefore, various approaches have been developed to increase their production and reduce their heterogeneity [31]. These approaches range from new cell culture devices to priming procedures, and most efforts have focused on 3 key approaches: improving MVB/ILV production, preventing degradation, and promoting exocytosis. Exosomes, which originate from early endosomes, undergo gradual maturation to form MVBs that contain numerous ILVs. Ultimately, mature MVBs either fuse with lysosomes for degradation or fuse with the plasma membrane, which results in their release into the extracellular space through exocytosis as exosomes [32, 33].

In this study, we used the Wnt agonist CHIR99021 to prime hucMSCs with the aim of enhancing the production of exosomes as well as their therapeutic efficacy. Our results indicated that pretreatment with CHIR99021 resulted in a significant increase in exosome secretion by hucMSCs, and exosome secretion in this group was approximately 1.5 times greater than that in the control group. This increase in exosome production was achieved without any changes in the characteristics of the hucMSCs or the size of the exosome particles. Furthermore, treatment with CHIR99021 enhanced the intracellular MVB and ILV contents in hucMSCs and promoted the exocytosis of MVBs. This process was partly mediated by SNAP25.

Several previous studies have suggested that activation of the Wnt signalling pathway can facilitate exocytosis in certain cell types, but their numbers are few, and the underlying mechanism is unclear. Zhang et al. discovered that the activation of β-catenin accelerated the assembly of the ESCRT complex and facilitated the exocytosis of 50-nm gold particles [19]. According to reports by Sorrenson et al., β-catenin promoted insulin secretion independently of its transcriptional coactivator TCF7L2; instead, β-catenin somehow facilitated the remodelling of actin and regulated the movement of insulin secretory vesicles towards the plasma membrane [20]. Sándor et al. reported that Wnt pathway activation was coupled with greater EV release in multiple organoid models [21], while Lu et al. suggested that Wnt signalling pathway agonists might exert the opposite effects on EV secretion [22]. In our study, a significant increase in exosome exocytosis was observed in hucMSCs after the Wnt signalling pathway was activated. Further transcriptomics led us to focus on cytosolic exocytosis and SNAP25, which is a crucial component of the SNARE complex. Through the siRNA-mediated knockdown of SNAP25, the CHIR99021-induced increase in exosomal exocytosis was partially abolished, confirming that Wnt agonists might partly rely on SNAP25 to facilitate exocytosis. These results proved that CHIR99021 could be a safe approach for enhancing exocytosis by hucMSCs, thus effectively increasing exosome production. For MVB/ILV production, we observed an increase in the MVB and ILV contents by TEM. Wnt signalling may promote exosome exocytosis and biogenesis; however, the expression levels of the core proteins of the ESCRT complex, namely, ALIX and TSG101, did not significantly change after CHIR99021 treatment. Combined with the decrease in LAMP2-CD63 costaining observed by confocal microscopy, these findings suggested that Wnt agonists increase the MVB and ILV contents by suppressing lysosome-mediated degradation. However, we cannot exclude the possibility that Wnt agonists might regulate the generation of MVBs and ILVs through mechanisms that are independent of ALIX and TSG101 protein synthesis. Other possible pathways include ESCRT-independent pathways, which are facilitated by the generation of ceramide or phosphatidic acid or by lipid raft formation in a Rab31-flotillin-dependent manner [34]. We will explore this topic in our future research.

Next, we explored the effect of CHIR99021 on exosome cargo loading and clinical applications. Proteomic analysis was used to identify the proteins whose levels were altered after Wnt signalling activation, and the proteins that were significantly upregulated in CHIR99021-treated exosomes were associated with pro-healing and cell migration processes. Previous research has demonstrated that activation of the nonclassical Wnt pathway induces the aggregation of IL-6, VEGF, and MMP2 into EVs in melanoma cells. Another study reported that activation of the classical Wnt pathway also promoted the aggregation of miRNAs into exosomes [35]. In our investigation, we observed an increase in the aggregation of TGFβ2, TGFβ3, AGO2, CCAR1, and other pro-healing proteins into exosomes following activation of the classical Wnt pathway. These findings collectively suggested that Wnt signalling has an impact on the sorting of EV contents. However, the specific mechanism underlying this phenomenon is not fully understood and warrants further exploration. For clinical applications, we used a full-thickness cutaneous wound model in diabetic mice, as well as NIH/3T3 fibroblasts, to observe whether the enhanced exosomes had better efficacy. Consistent with previous reports [25, 36, 37], control exosomes from untreated hucMSCs significantly accelerated the wound healing procedure, and our CHIR-Exos were even more effective: compared with control exosomes, CHIR-Exos significantly accelerated the wound healing rate, enhanced local collagen deposition, promoted angiogenesis, and reduced chronic inflammation in diabetic mice. Subsequent in vitro experiments confirmed that the CHIR-Exos promoted wound healing by facilitating cell migration, inhibiting oxidative stress-induced apoptosis, and preventing cell cycle arrest. All of these results suggested that pretreatment with CHIR99021 not only promoted hucMSC-derived Exo production but also improved the therapeutic efficacy of these particles, thus providing a potential approach for clinical application.

In summary, our findings indicated that CHIR99021 could be a safe approach for facilitating exosomal secretion by hucMSCs and augmenting the loading of pro-healing proteins into exosomes to promote diabetic wound healing. However, before we apply these results to biopharmaceutical development and commercial mass production, several challenges need to be addressed. First, although we found that CHIR99021 delayed the passage-induced senescence of hucMSCs, the possibility that exosome cargoes change during continuous subculture still existed. Therefore, exosomal proteomic comparisons are necessary after continuous passaging. Second, the influence of the Wnt signalling pathway on exosomal protein cargo loading and its specific mechanism remain unclear and warrant further exploration. Finally, the precise impact of CHIR99021 on ILV production and its underlying mechanism remain unclear, so further investigations are necessary, particularly regarding the potential involvement of the ESCRT-independent pathway. We hope to address these limitations in our future research.

## Conclusion

In this study, we investigated the potential of a Wnt agonist for priming hucMSCs to enhance exosome secretion and improve therapeutic efficacy in diabetic wound healing. Pretreatment with the Wnt agonist CHIR99021 significantly enhanced exosome secretion by hucMSCs. Mechanistically, CHIR99021 promoted exosome secretion by facilitating exocytosis of MVBs, which was partially mediated by SNAP25. Furthermore, CHIR99021-primed exosomes exhibited notably enriched levels of proteins associated with cell migration and wound healing. Consequently, compared with the administration of exosomes from untreated hucMSCs, the administration of these exosomes led to a significant improvement in diabetic wound closure. In conclusion, these findings demonstrated that CHIR99021 pretreatment effectively enhances both exosome production and cargo loading in hucMSCs, leading to exosomes with superior therapeutic potential for diabetic wound healing. This strategy offers a promising, safe, and cost-effective approach to optimize Exo-based therapy for diabetic ulcers, paving the way for further clinical translation.

### Electronic supplementary material

Below is the link to the electronic supplementary material.

Supplementary Material 1

## Data Availability

All data and materials are available in the manuscript.

## Abbreviations

hucMSCs: Human umbilical cord mesenchymal stem cells
MSC: Mesenchymal stem cells
EV: Extracellular vesicles
Exo: Exosome
MVB: Multivesicular bodies
ILV: Intraluminal vesicles
STZ: Streptozotocin
TEM: Transmission electron microscopy
NTA: Nanoparticle tracking analysis
AChE: Acetylcholinesterase
KEGG: Kyoto Encyclopedia of Gene and Genomes
GO: Gene Ontology
CTRL-Exo: Control exosome
CHIR-Exo: CHIR 99021-pretreated exosome
XAV-Exo: XAV939-pretreated exosome
ELISA: Enzyme-linked immunosorbent assay
