# Supplementary material for: Activation of the Wnt/β-catenin signalling pathway enhances exosome production by hucMSCs and improves their capability to promote diabetic wound healing
Source: J Nanobiotechnology. 2024 Jun 26;22:373. doi: 10.1186/s12951-024-02650-x (PMC11201861; doi:10.1186/s12951-024-02650-x)
Supplement: Supplementary file 1 — Supplementary Material 1 [file 12951_2024_2650_MOESM1_ESM.docx]

**supplementary material**

Figure S1

(A) Detection of cellular senescence by β- galactosidase staining. (B) Western blot analysis of LAMP2, STXBP1, VAMP2, VAMP3, TSG101, ALIX and HSP70 in each group. (C) Location of Rab7a was observed using immunofluorescence after treatment of hucMSCs with Wnt signaling pathway agonists and inhibitors. (D) Internalization of exosomes was observed using fluorescence microscopy in each group.

**
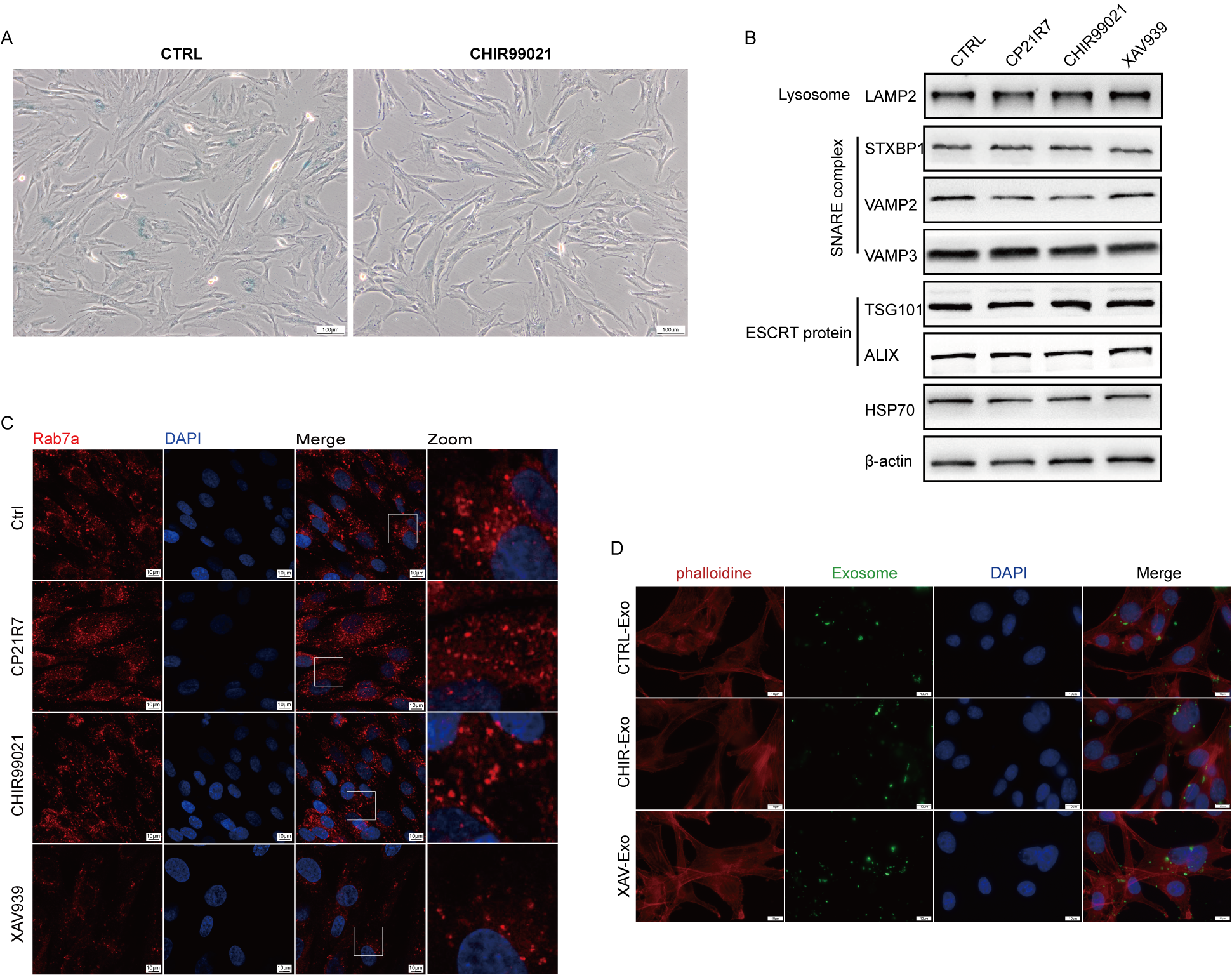
**
